# American Medical Association

**Published:** 1885-07

**Authors:** 


### AMERICAN MEDICAL ASSOCIATION.

### SECTION OF DENTAL AND ORAL SURGERY.

REPORTED FOR THE INDEPENDENT PRACTITIONER BY "MRS. M. W. J."

(Concluded from page 330.)

### SECOND DAY—AFTERNOON SESSION.

Oscar J. Coskery, M. D., of Baltimore, read a very valuable paper, entitled "A Case of Sarcoma of the Lower Jaw, with Successful Removal."

Before reading the paper Dr. Coskery made some explanatory remarks, and exhibited a cast of the left side of the face and neck, as before the operation, a cast of the jaws showing the articulation of the teeth (which were very much displaced by the growth of the tumor, with immense enlargement of the lower jaw), the jawbone itself as removed with the tumor, photographs of microscopic sections of the Sarcoma tissue, etc. The paper was simply a record of the case, with explanatory notes, as follows:

The subject was Pierre King, a colored boy, fifteen years old, a native of Maryland. His mother had, for several years, noticed a gradual enlargement of the left side of the lower jaw, the teeth becoming more and more displaced. A sudden and rapid enlargement of the gland under the jawbone frightened the boy, and he applied for admission to the hospital.

The inferior maxilla was found to be enormously enlarged, the teeth displaced in all directions, the gland very much enlarged, the tongue crowded over into a narrow groove on the right, and the oral cavity much depressed. He was admitted to the hospital on the 1st of May, 1882. On the 15th of May he was chloroformed and the operation performed. An incision of the soft tissues was made in the median line of the lower lip to below the chin, thence over the most prominent portion of the tumor outwardly to the angle of the jaw, thence upward. The flap was dissected off and turned up, exposing the growth. The facial artery at this point of the operation was cut and ligated. The right central incisor was next extracted, and from that point the bone was sawed through. The soft connective tissues were next severed, and, with strong traction, the neck of the tumor gave way. With forceps an attempt was made to tear the whole mass out, but the jawbone broke. The bone forceps were finally used to wrench it out, which was finally done with no very great hemorrhage. In cutting out the enlarged gland, profuse venous bleeding followed, and it was found that a branch of the external jugular had been cut. This was ligated at both ends, and the flap replaced in position and covered with dry lint, with a bandage over all. The boy did well from first to last; the cut healed kindly, Listerine being used throughout the whole course of treatment to keep the wound aseptic. He was discharged from the hospital twenty-one days after the operation, with only one point of suppuration left for drainage.

A microscopical examination by Dr. Aug. Hoen showed the tissue to be a dense, white fibrous stroma, with dilatations filled with spindle-cells, closely packed in some portions, quite loosely in others, each cell having a single oval nucleus. The photographs show microscopic sections cut on different planes. The cells constitute true fibroid sarcoma, with no tendency to cystic degeneration. Dr. Coskery saw the boy two years after the operation, but failed in his efforts to obtain a photograph. He is employed as a farm-hand, and supports himself. He masticates quite comfortably, and has had no trouble since his discharge from the hospital. There has been no reproduction of bone, but the anterior portion of the lower jaw has been drawn towards the vacant space.

*Dr. J. L. Williams*—How frequent are cases of this character?

*Dr. Coskery*—Epulis involving the lower jaw, or fibroid tumors seemingly non-malignant, commencing in the jawbone itself and not in the gum, are very seldom met with. In this case there was no tendency to degeneration, or to a cystic form. This particular form is exceedingly rare; indeed, it is scarcely mentioned by authors.

*Dr. Baldwin*—The thanks of the section are due to Dr. Coskery for his valuable paper. It will probably be many years before a similar case will come before us. I would like to inquire as to the probable permanency of cure. Its doing well two years later was fairly conclusive that the patient had entirely recovered, with no probability of recurrence. I should, however, feel suspicious of possible sarcomatous infiltration in remaining portions. It is very important to guard against the desire of saving too much tissue. We must cut boldly and be sure to reach healthy tissue.

*Dr. Williams*—In this case the operation would appear to have gone quite beyond the range of disease.

*Dr. Baldwin*—We are prone to try and save as much as possible, but nature will reproduce healthy tissues more readily than she will remove diseased tissue.

*Dr. Coskery*—There is, of course, danger of recurrent fibre in fibroid sarcoma. All of the bone involved must be removed, as was done in this case, from the median line to the condyle. In a case of sarcoma of the chest, I operated seven times for recurrence, and the patient finally died with a seven pound sarcoma. But in the case here recorded I did not expect a recurrence. It is useless to remove only part of the affected bone, but the lower jaw being in two halves, in removing from the median line to the condyle the entire bone was removed.

Dr. J. R. Walker exhibited some casts of remarkable cases of irregularity, explaining the methods employed in regulating, with casts of the teeth after the operation. Also, what is probably the youngest tooth ever filled, a superior temporary central incisor, first filled when the child was eleven months old, and the tooth but two months erupted. At the age of five years the tooth was refilled with gold. The tooth erupted with a yellow spot on the labial surface, which soon developed into a cavity of decay. The permanent tooth which replaced it is marked with a similar, but white spot, which, however, has not decayed, the boy being now eighteen years old.

Discussion of the paper read by Dr. Williams at the first session was declared in order. The following is the argument of the paper, the title being "The Alternation of Rest with Effort."

The paper was prefaced by the remark that, when a student, he heard old Dr. John C. Warren speak of the grateful relief afforded the eyes, when fatigued from long continued effort in surgical operations, by the momentary rest afforded by simply raising them and letting them rest on some object in a distant part of the room, or, if the operation would permit, of stepping to the window for a moment.

More recently, an eminent American oculist has written of the great injury done by continuous application of the eyes after they become fatigued. The same is equally true of all the other organs of the body. The idea represents a principle. The organs need rest. The old idea that "exercise strengthens," without any other consideration, that the longer we exert them the stronger they grow, represents a false principle. On the contrary, fatigue to exhaustion produces weakening and debility. It is the same with all occupations and with all the organs. The student, the professional man, the oarsman, the pedestrian, may exert himself continuously, till suddenly he finds that his strength, whether mental or physical, is gone forever. Too long and too continuous labor in any one department will shatter the nerves, and utter prostration will follow, or the individual may, as has been the case, fall dead at his post. In our own department we must remember, too, that we exhaust others as well as ourselves. We must recognize the fact that we too often submit our patients to too long and too continuous fatigue, and that days and weeks may be required to recover from the effect, even if more serious results do not follow. The danger is not in the amount of fatigue endured, nor in the severity of the exercise, but in the long continuance of labor after fatigue is felt, passing beyond the point of fatigue to that of exhaustion, either of some particular organ, or of the entire system. The point urged is that, if possible, a man ought to rest just when he is tired. Alternate labor with rest, and, at the same time, let our patients do the same thing.

*Dr. Walker*—The subject is very important and the advice very valuable, but it is exceedingly difficult to heed.

There are different classes of men in our profession. One class works only for money; they do not get over-fatigued and their operations are not tedious; they go through a patient in a hurry, but they make every moment pay well. There is another class whose interest in humanity and in science outweighs the desire for pelf. This class of men continually overwork. They need advice, but they seem unable to heed it. They are usually men of strong sympathies. In their desire for success, and in their sympathy for their patients, they wear themselves out, and they die poor. They cannot find time to make money, nor take time to rest.

*Dr. Baldwin*—In my studies as a medical student I gave particular attention to the eye and its diseases. The principle laid down in the paper is specially applicable to the dentist. We are obliged to work in a very strong light, at a very short focus, for a very considerable length of time. These conditions are trying to the eyes, and we consequently find very general complaint among dentists of trouble with the sight. This is very often attributed to the direction from which the light comes, a subject which is much discussed in our societies, there being a great difference in opinion on this as on all other subjects. I believe, however, that this occasional brief rest for the eye will be found very beneficial.

*Dr. Williams*—I have, for many years, been in the habit of occasionally looking away from my work, letting the eye rest for a moment on some grateful object, and believe that the result proves the practical utility of the principle. I have no weakness of the eyes, and no irritation—only the change natural to increasing years. It is simply obeying a rule of nature. A child when tired with running, naturally sits down to rest the limbs. The same principle applies to the eyes.

*Dr. Marshall*—No men tax their eyes more than the dentists, and none have such poor eyes, though all do not know it. I was born with astigmatism, but was not aware of it. For years I suffered with very severe headaches ; attributed it to the stomach, but could obtain no relief. Happening to be in the office of an optician, I accidentally noticed, in looking at the radiating lines of a circle, that the horizontal lines appeared blurred; they looked gray instead of black. I then had my eyes thoroughly examined, and found that I had used and abused them all my life. The only wonder was that I had not suffered from severe inflammation. I had my eyes fitted with suitable glasses, and have worn them ever since, taking them off only when I go to bed. As the result, instead of having headaches two and three times a week, I have it perhaps only once in two months. The glasses afford artificial rest. I had attributed my trouble to overwork, and to my chair; I then used an old Archer chair which I changed for an S. S. White, pedal lever; but though my general health improved from better conditions for work, my headaches still continued until I got the right glasses. I also obtain great relief from the application of the principle laid down in the paper read. The eyes of nine out of ten dentists, if examined by a competent oculist, would be found defective from constant application to fine work at an unnatural focus, often twelve inches when it should be eighteen. It is a constant strain on the muscles. The headaches of the dentists can generally be attributed to the eyes.

*Dr. Williams*—There must also be a true understanding of the meaning of the word *rest.* It is commonly supposed that a change of occupation is rest; that is not my idea. A change is simply refreshing, but rest is dispensing with all application. If we look straight at one object a long time, and then look at another, the eye does not rest, though it is refreshed. Let the eyes revolve slowly, not looking at anything; that is resting them. If a man gets very tired walking up hill, it will refresh him to turn and walk the other way, but it will not rest him. He must stop walking entirely, to really rest. It must be a discontinuance, not a change of application. Effort in any direction draws from the stock of vital power. The stock is diminished if the exercise is continued, even in a different direction. Repose, not change, is rest. If a man works at the chair all day, it is refreshing to change to laboratory work at night; he feels refreshed by the change, but it does not rest him. He is drawing on his vitality all the time. He must have absolute cessation in order to replenish or accumulate vital power. A man is more easily fatigued in the early hours than later in the day. If a man commences gently, he will accomplish more than if he takes his most difficult operations at the beginning of the day. His day's work should begin like the sunrise.

*Dr. Walker*—Repose is not always rest. Rest consists in building up, in recuperation, in the proper distribution of nutrition. If a man stands in one position for hours, he exhausts his nerve force; there is no circulation of blood in certain portions of the system. Repose in such conditions would not afford rest; the blood requires circulation by active exercise. Under those circumstances a man might lie like a log all night, and not be at all refreshed or invigorated by his night's sleep. Stirring up the blood would be rest in that case. In the use of the eyes it is a great mistake to work in a varying light, exposed to changing shadows, or with glaring sidelights. After an experience of thirty-five years I have learned to avoid anything but a direct light upon my work. I remove everything that produces a glare, have the walls a neutral tint, and all white objects out of range of vision. The light must also be under control as it changes with the hours of the day. The vision is confused and the eyes injured by glare from bright objects, or by cross lights or side lights. We want a steady, direct light upon our work.

*Dr. Williams*—This matter of glare has not received sufficient consideration. Too great a flood of light obtunds the sensitive optic nerve. With a bright south light the optic nerve loses its strength early.

The student, when weary of study after long hours, goes out and takes vigorous exercise, say in rowing; he feels refreshed, and his brain is rested, but we must remember that men who study exhaustively have not an inexhaustible supply of vital force. They must have absolute freedom from application. A barrel is soon emptied if it is running from a faucet at both ends. If only the arm is tired, rest the arm; but if fatigue is general, the rest must be absolute.

*Dr. Baldwin*—The scope of the paper is wider than that taken in the discussion. We must consider the point not only of rest for the operator, but of rest for the patient. When partially exhausted we become nervous, irritable. The patient becomes the same; he is unable to control certain physical motions; the effort required for the necessary restraint becomes unbearable sometimes; we will call it nervousness. When you feel that way, and when you realize that your patient feels that way, a dose of from thirty to sixty grains of bromide of potassium, in solution, will be found invaluable. Before undertaking a long and difficult operation, if both patient and operator would prepare themselves by taking from twenty to forty grains of bromide, in solution, they would be astonished at the augmented powers of endurance. There will be less exhaustion, and a hard sitting will be endured with freedom and ease.

*Dr. Walker*—We should all study to avoid extremes. The brain is rested by exercising the muscles, but neither brain nor muscles should be exercised to exhaustion. When at college I had a fellow-student whose worldly circumstances and physical health were both apparently very poor. He boarded at a water-cure, five miles from the college, walking back and forth night and morning, supporting himself by work out of hours. While others were poring over their books with exhausted brain, and lessons always poorly committed, this young man was recuperating his mental powers by physical exercise, and his lessons were always well prepared.

*Dr. Smith*—In the use of the eyes most of us are governed by individual strength and habits. Some will feel no fatigue where others would be exhausted. Before I was advised to get glasses I had no trouble with my eyes, but since I took to glasses my eyes get fatigued. Glasses afford only one focus, and the eyes get tired. I find them a hindrance to work. The proper management of light is a most important point. The changes of light from shadows and clouds are bad. We need direct light from above, with no glass, and no change in the degree of light. A person in good health, with eyes of ordinary strength, will not feel the need of rest for the eyes until the whole body is fatigued. If in reading fine print the lines blur and run together, then it is time to stop. I doubt if dentistry is good for the eyes. We work at a closer focus than is natural.

*Dr. Williams*—We must not be afraid to use helps. We need lenses for small objects as much as the jeweler or the engraver. It is a false sensitiveness to fear that patients will think your eyes poor. Many, from this fear, use reflecting mirrors, or a magnifying glass. A lens is much better. We must have rest, and also use helps, no matter what remarks patients may make. They might also think that because we use absolute alcohol in the palm of the hand we are therefore drunkards. The objective point in our work requires magnifying.

The discussion was then closed.

Dr. J. S. Marshall, of Chicago, Ill., was nominated for Chairman of the Section, and Dr. A. E. Baldwin, of Chicago, Ills., for Secretary.

The time and place of the next meeting, by the action of the American Medical Association, was fixed for St. Louis, on the first Tuesday in May, 1886.

The Section adjourned to meet at that time and place.

